# Prevalence of Noise-Induced Hearing Loss among Textile Industry Workers in Dar es Salaam, Tanzania

**DOI:** 10.5334/aogh.2352

**Published:** 2019-06-17

**Authors:** Zephania Abraham, Enica Massawe, Daudi Ntunaguzi, Aveline Kahinga, Shaban Mawala

**Affiliations:** 1 Department of Surgery-University of Dodoma, College of Health Sciences, TZ; 2 Department of Otorhinolaryngology, Muhimbili University of Health and Allied Sciences, Dar es Salaam, TZ; 3 Department of Otorhinolaryngology-Muhimbili National Hospital, Dar es Salaam, TZ

## Abstract

**Background:**

Noise-induced hearing loss (NIHL) is the most common preventable cause of deafness. The degree of NIHL is determined by intensity, duration of exposure, spectral characteristics of the noise, and individual susceptibility. Industrial workers in both developed and developing countries are all at risk of suffering from NIHL.

**Methods:**

An industry-based descriptive cross-sectional study was conducted. Data was collected using structured questionnaires, where sound intensity and hearing assessment were measured using a portable sound level meter and a portable audiometer, respectively. Data was analyzed using Statistical Package for Social Sciences (SPSS) program version 21.

**Findings:**

Out of 265 industrial workers who were recruited in this study, 161 (60.8%) were males and the predominant age group was 22 to 35 years (43%). The prevalence of NIHL was found to be 58.5%. Of 155 workers with NIHL, 101 (67.7%) were males. The most common age group was greater than 49 years. Of those with work experience from 5 to 10 years, only 38.5% had NIHL. The most common symptom was hearing loss, accounting for 24.9% of cases.

**Conclusion:**

The overall prevalence of NIHL was higher in textile industry workers. The prevalence was higher in males, older workers, and those who experienced prolonged exposure.

## Introduction

Noise-induced hearing loss is a sensorineural hearing deficit that begins at the higher frequencies (3,000 to 6,000 Hz) and develops gradually as a result of chronic exposure to excessive sound levels [1].

Although the loss is typically symmetric, noise from such sources as firearms or sirens may produce an asymmetric loss. Hearing loss caused by exposure to recreational and occupational noise results in devastating disability that is virtually 100 percent preventable [2].

Noise-induced hearing loss is the second most common form of sensorineural hearing deficit after presbycusis (age-related hearing loss). Shearing forces caused by any sound have an impact on the stereocilia of the hair cells of the basilar membrane of the cochlea; when excessive, these forces can cause cell death [2].

Worldwide, the burden attributed to occupational noise ranges from 7% in western countries to 21% in developing countries (average 16%) [3]. Prevalence of NIHL in industrial populations varies by industry (electrical workers, sand and gravel workers, and construction workers), and it lies between 37% and 59.7% [4].

A study done in America in a construction company reported 60% of operating engineers had NIHL, and a higher rate was observed among workers who reported longer years of working. Thirty-eight percent (38%) reported tinnitus, and 62% reported difficulty understanding what people said when speaking loudly [5].

Studies from other parts of the world, such as Sweden, depicted a NIHL prevalence of 22%, and a study from South Africa found approximately 73.2% of miners in the industry to be exposed to noise levels above the legislated occupational exposure limit of 85 dBA [6].

A study done in Dar es Salaam revealed the minimum peak noise level was 87 dBA, and the maximum was 116.5 dBA, with an average of 92.6 dBA. Of those investigated fully, 50.8% and 46.5% in area A and B, respectively, were found to have audiogram patterns typical of NIHL. The study revealed 22.5% and 18.6% of employees had permanent threshold shifts (PTS) in area A and B, respectively, while 28.2% and 27.9% had temporary threshold shifts (TTS) in area A and B, respectively. The study showed significant NIHL in the studied population [7].

Despite the existing evidence of NIHL in industrial workers, data on NIHL in Southern and East Africa remains scarce. The aim of this study was thus to determine the magnitude of NIHL among textile industry workers in Dar es Salaam, Tanzania, creating a basis for limited data in Sub-Saharan Africa.

## Materials and Methods

### Study design and participants

This was an industry-based descriptive cross-sectional study conducted from May to December 2014, and it included all workers 60 years old or younger and meeting the inclusion criteria.

### Sampling method

Convenience sampling technique was utilized with selection based on the most available sample. Workers who met the inclusion criteria were chosen provided they were available during data collection, and they were added until the desired sample size was achieved.

### Inclusion and exclusion criteria

Workers in the production department who were 60 years old or younger, those who had a normal hearing assessment status at the time of employment, those who were in a noise-free zone for 16 hours or more, and those with at least five years of exposure to noise were set as the inclusion criteria.

Workers with ear infections and those with other obvious causes of hearing loss apart from excessive noise (e.g., those exposed to ototoxic drugs or explosives and those with cerebellopontine angle tumors) were excluded from participating in the study.

### Sample size

A total of 265 industrial workers were recruited as the desired sample size.

### Data collection methods

Structured questionnaires were used to collect data from the desired participants. Hearing assessments were performed by qualified audiologists. Hearing assessments were done in a room with the lowest sound intensity as much as possible (at most 35dBA).

### Data analysis

Data analysis was done using SPSS version 21. Statistical association was computed using cross tabulations, and a Chi-square test was used to compare proportions. P value of <0.05 was considered statistically significant.

### Ethical considerations

Workers were provided with information about the study and then asked to provide written consent to participate. They were free to discontinue participation at any point. Ethical approval was provided by the Research and Publication Committee of the Muhimbili University of Health and Allied Sciences (MUHAS).

## Results

### Demographic characteristics of the study population

From May to December 2014, 255 textile industry workers were recruited. A majority (114, 43%) were between the ages of 22 to 35. The mean age was 40.28 (SD = 12.6), the minimum age was 22 years, and the maximum age was 60 years. Male predominance (161, 60.8%) was found in this study. There were more males than females in all age groups except 36 to 49 years, where there were 52.4% females (X^2^ = 20.1, p-value < 0.001) (Table 1).

**Table 1:** Age and sex distribution of study participants from the textile industry.

Age (years)	Sex		Total	Chi-square, p-value

	Male	Female		

	Frequency(%)	Frequency(%)	n (%)	

22 to 35	61 (53.5)	53 (46.5)	114 (43)	
36 to 49	30 (47.6)	33 (52.4)	63 (23.8)	20.1, <0.001
>49	70 (79.5)	18 (20.5)	88 (33.2)	
**Total**	161 (60.8)	104 (39.2)	265 (100)	

### Working experience of study participants

**Figure 1:**
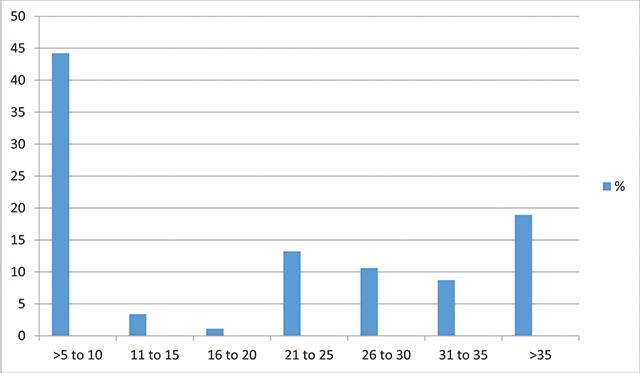
Work experience of the study participants.

The textile industry had a large number of workers (117, 44.2%) with work experience of 5 to 10 years. The lowest number of workers had work experience of 16 to 20 years (3, 1.1%) (Figure 1).

### Distribution of workers in the textile industry

A majority of workers (213, 80.3%) enrolled in the study were in the loom shade department. The loom shade department is the noisiest, with a sound intensity over 95 dB. Drawing frame and finishing were the only departments with a safe sound intensity of 77 to 85 dB. All workers reported working more than 8 hours per day, and none reported using hearing protective devices (Table 2).

**Table 2:** Distribution of textile industry workers.

Department (sound intensity in dB)	Frequency(n)	Percent (%)

Drawing frame (77)	23	8.7
Finishing (81–85)	16	6
Loom shade (95–100)	213	80.3
Preparation (85–87)	11	4
Sizing (85–87)	2	1
**Total**	265	100

### The prevalence of NIHL among textile industry workers

**Figure 2:**
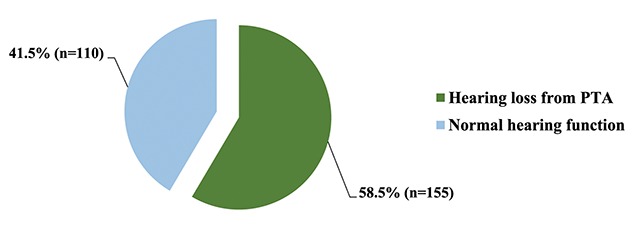
The prevalence of NIHL among textile industry workers.

There were 155 workers (58.5%) with NIHL in the textile industry (Figure 2).

### Prevalence of NIHL among textile industry workers by age and sex

The prevalence of NIHL among textile industry workers increased significantly with age (X^2^ = 29.3, p-value < 0.001), and the most affected age group was more than 49 years old (n = 68, 77.3%). By sex, 101 (62.7%) males were affected compared to 54 (51.9%) females, but this was not statistically significant (X^2^ = 3.04, p-value = 0.08) (Table 3).

**Table 3:** The prevalence of NIHL among textile industry workers by age and sex.

Specific variable	Have hearing loss	Normal hearing	X^2^, p-value

**Age (years)**			
22 to 35	46 (40.4)	68 (59.6)	
36 to 49	41 (65.1)	22 (34.9)	
More than 49	68 (77.3)	20 (22.7)	29.3, <0.001
**Sex**			
Male	101 (62.7)	60 (37.3)	
Female	54 (51.9)	50 (48.1)	3.04, 0.081
**Total**	155 (58.5)	110 (41.5)	

### The prevalence of NIHL among textile industry workers by duration of exposure to excessive noise

**Figure 3:**
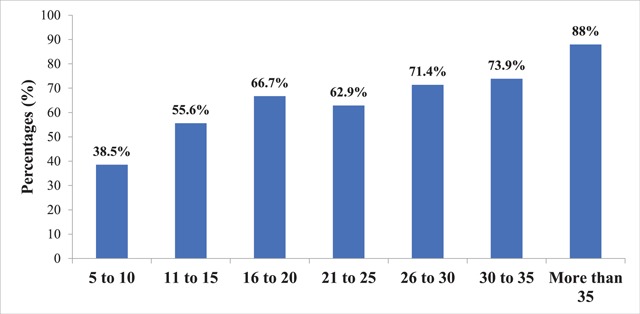
The prevalence of NIHL among textile industry workers by duration of exposure to excessive noise.

The prevalence of NIHL among textile industry workers significantly increased as the duration of working increase (X^2^ = 41.84, p-value <0.001). Those who worked for more than 35 years (88%) had NIHL, compared to those who worked for 5 to 10 years (38.5%) (Figure 3).

### Distribution of hearing loss laterality, notch presence, side of notch, and frequency at which notch is present

A majority of workers (107, 69%) had hearing loss in both ears, notch presence was present in 90 (58.1%) workers, 40 (44.4%) had the notch in both ears, and the most common frequency demonstrating the notch present was 4000Hz for 43 (47.7%) workers (Table 4).

**Table 4:** Frequency distribution table showing lateralization of hearing loss, notch presence, side of notch, and frequency at which notch is present.

Item	Frequency (n)	Percent (%)

**Side of hearing loss**		
Left	26	16.7
Right	22	14.2
Both	107	69.1
**Presence of notch**		
Yes	90	58.1
No	65	41.9
**Side of notch**		
Right	21	23.3
Left	29	32.3
Both	40	44.4
**Frequency at which notch present**		
3000Hz	9	10
4000Hz	43	47.7
6000Hz	38	42.3

### Common symptoms reported by textile industry workers

**Figure 4:**
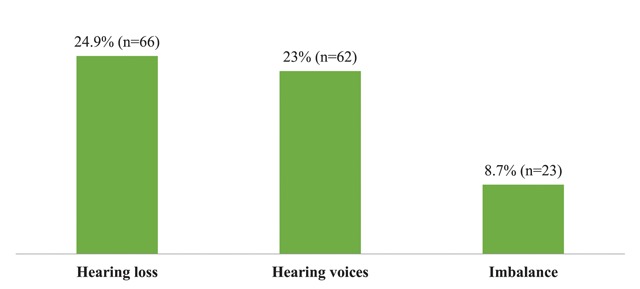
Common symptoms reported by textile industry workers.

Hearing loss was reported in 66 (24.9%) workers, tinnitus in 62 (23%) workers, and imbalance in 23 (8.7%) workers (Figure 4).

## Discussion

The aim of this study was to determine the magnitude of NIHL among textile industry workers in Dar es Salaam, Tanzania. The prevalence of NIHL among textile industry workers was found to be 58.5%. Worldwide, the prevalence was within the range of 37% to 59.7% [3]. Our results are higher than those found in the study done in Dar es Salaam by Moshi and Minja, where the audiogram patterns typical of NIHL were 50.8% and 46.5% in area A and B, respectively [7]. The increased prevalence may be due to the nature of employment offered by the textile industry, where workers are permanently employed, work for over eight hours per day, five days per week, and over 85% of workers are exposed to sound intensity over 85dB and none use hearing protective devices.

This study revealed 69.1% of the workers have bilateral NIHL, with right ear predominance afflicted by NIHL. This finding is similar to other studies [5]. This may be due to uniform distribution of excessive noise in the working environment leading to a large number of bilateral NIHL.

This study also revealed 58.1% of workers with NIHL had notch, 44.4% in both ears and 47.7% at 4000 Hz, followed by 42.3% at 6000 Hz. This is different from a study done in musicians in the United States, where notch was in 45%, and 78% had notch at 6000 Hz, 22% at 4000 Hz, and 2% at 3000 Hz [8]. This may be due to different excessive noise exposure.

This study revealed that prevalence of NIHL was increasing with age: 40.4% in age group 22 to 35 years, 65.1% in age group 36 to 49 years, and 77.3% in age group more than 40 years.

This finding is similar to a study done in Sweden, which revealed the prevalence of NIHL to be 50% in age group 35 to 39 years and 90% in age group 55 to 59 years. In a study done in the United States among operating engineers, NIHL was 75% among workers in their forties and 100% in workers 50 and 60 years old [5]. Another study done in the United States revealed the prevalence of NIHL was 8.5% in the third decade, 17% in the fourth decase, and almost 100% in the sixth decade [9].

Based on the previously mentioned studies, age and excessive noise may be two separate causes of hearing loss or the two (age and excessive noise) could cause hearing loss by synergism.

The study revealed the prevalence of NIHL based on sex to be 62.5% in males. Such male predominance appears to correlate with findings from other studies [5]. This may be due to the way jobs are distributed in industries, where men are operating machines that produce more hazardous sounds and women are in less noisy environments. This may explain the large percentage of NIHL in males.

This study revealed the prevalence of NIHL was increasing as the duration of exposure to excessive noise increased. The prevalence of NIHL based on work experience was 38.5% in those with 5 to 10 years of work experience, 55.6% in those with 11 to 15 years of work experience, 66.7% in those with 16 to 20 years of work experience, 62.9% in those with 21 to 25 years of work experiene, 71.4% in those with 26 to 30 years of work experience, 73.9% in those with 31 to 35 years of work experience, and 88% in those with more than 35 years of work experience. The pattern is similar to a study done in the United States that found the prevalence of NIHL was 75% for workers with 20 to 29 years, 89% for workers with 30 to 39 years, and 100% for workers with over 40 years in construction [9].

This study revealed the common symptoms reported to be hearing loss (24.9%), tinnitus (23%), and imbalance (8.7%). This pattern is similar to the study done in the United States in which operating engineers reported hearing loss (62%) and tinnitus (38%) [5]. In the present study, the proportions were lower. This may be due to the presence of more excessive noise in the U.S. construction industry.

In a study done in musicians, the most common symptom was tinnitus, followed by hearing loss (90% of musicians reported tinnitus), and a majority presented with hyperacusis [10]. Hyperacusis was not reported in this study, neither in most studies with industrial workers as their basis.

## Conclusion

The overall prevalence of NIHL was higher in textile industry workers. This calls for the need to provide protective gear to workers in stations generating excessive noise. Moreover, the prevalence was higher in males, older workers, and those experiencing prolonged exposure.
